# Magnetoelectric oxide based stochastic spin device towards solving combinatorial optimization problems

**DOI:** 10.1038/s41598-017-11732-w

**Published:** 2017-09-12

**Authors:** Saima Sharmin, Yong Shim, Kaushik Roy

**Affiliations:** Purdue University, School of Electrical & Computer Engineering, West Lafayette, IN 47907 USA

## Abstract

Solving combinatorial optimization problems is challenging. Mapping onto the ground-state search problem of the Ising Hamiltonian is a promising approach in this field, where the components of the optimization set are modeled as artificial spin units. The search for a suitable physical system to realize these spin units is an active area of research. In this work, we have demonstrated a scheme to model the Ising Hamiltonian with multiferroic oxide/nanomagnet units. Although nanomagnet-based implementation has been shown before, we have utilized the magnetoelectric effect of the multiferroics to make voltagecontrolled spin units with less current flow in the network. Moreover, we have proposed a unique approach of configuring the coupling network of the system directly from the Ising Hamiltonian of a traveling salesman problem (TSP). We have developed a coupled micromagnetic simulation framework and solved TSPs of size 26-city and 15-city with an accuracy of 100% for the latter.

## Introduction

The solution of a wide variety of tasks in modern world, ranging from tour planning, image processing to integrated circuit design, are based on the idea of combinatorial optimization^1^. These tasks, in general, involve finding a combination of objects/states from a large collection of possible outcomes. Depending on the depth of computational complexity, many of these problems are categorized as nondeterministic polynomial (NP)-hard or NP-complete. Finding a globally optimal solution for these problems is difficult, in some cases, impossible, by modern computers, as the time requirement for computation grows exponentially or worse with the size of the input. Hence, extensive research is going on in search of efficient schemes to find optimal or near-optimal solutions.

Ising model^2^, due to its combinatorial interpretation, has attracted growing interest as a tool to mathematically formulate many combinatorial optimization problems. They can be mapped as the ground-state search problem of the Ising Hamiltonian (explained in the next section). As a result, numerous algorithms and systems have been proposed and demonstrated to solve the Ising model. Simulated annealing^3^ is one of the oldest approaches pursued in this field. Recently, quantum annealing based systems (D-wave machine^4^) have been demonstrated to solve various optimization problems like protein folding^5^, graph isomorphism^6^ etc. Ising spin chip^7^ based on CMOS architecture and coherent Ising machine^8^ based on laser networks are newer additions to this family. In recent times, nanomagnet-based implementation of the Ising model has been proposed in refs 9 and 10, where spin Hall material/stochastic nanomagnets are used as a physical model of the artificial spin units. On the other hand, in this work, we have introduced a stochastic spin device consisting of multiferroic oxide/nanomagnet stack as the fundamental spin unit. The difference in their mechanism of operation can be illustrated based on the three main components of each unit.

- Write (Manipulation of the spin states): In previous works^9, 10^, write unit consists of a spin Hall material/nanomagnet heterostructure, where the magnetization moment of these nanomagnets represent each spin state. Manipulation of these states is induced by the current flow through the spin Hall materials. Although the current requirement for individual stochastic unit is less than the conventional critical switching current of magnetic tunnel junctions^9^ (MTJ), the complete network consists of *N* ^2^ such units for an *N* city traveling salesman problem (TSP), leading to huge current flow through the whole network. For example, if *N* = 10, the total current requirement roughly amounts to 100 × *I*_*c*_, where *I*_*c*_ is the current through individual spin unit. This poses challenge to the scalability of the system as the problem size increases. On the other hand, in our device, the write unit consists of a multiferroic oxide/nanomagnet stack, where the magnetization state is controlled by the voltage applied across the oxide. This oxide is thick enough to inhibit current flow in all of the *N* ^2^ write units.
- Read: Reading the magnetization state in both methods are performed by a current flow through an MTJ, where the free layer is coupled to the nanomagnet of the write unit through dipolar interaction. However, the output parameter in previous works is current (proportional to the resistance of the MTJ), which is passed on to the inputs of interacting spin units. But, in our device, the MTJ current is converted to an output voltage by using a voltage divider circuit. This voltage later provides inputs to coupled units.
- Interconnect: The spin-spin interaction is emulated by sending the output from one spin unit to the other. The current-based input-outputs in previous works cause current flow from one interacting unit to the other, whereas, in our device, there is no current flow between connected spin units due to voltage-based coupling. This is an important aspect of scalability of the scheme. Also previous works suggest additional amplification^9^ or CMOS circuits^10^ in between coupled spin units in order to drive the fan-out, whereas in our device, direct cascading is possible by proper device engineering.

It is to be noted that the network of spins in our model does not entirely resemble a Boltzmann machine^11^. The focus of our scheme is to make the lower energy states to be more probable with no restriction on the overall energy landscape. Therefore, although the probability of observing a state with energy *E* gradually increases with decreasing *E*, it may or may not follow the Boltzmann distribution.

In this work, we have presented the structure and switching characteristics of these voltage-controlled spin units. Additionally, we have showed a way of direct configuration of the spin-spin and spin-external source coupling network from the Ising Hamiltonian, taking TSP as an example. As a validation of our model, we have developed a coupled LLG (Landau-Lifshitz-Gilbert) equation-based simulation framework and solved TSP problems of size 15-city ((*N* − 1)^2^ = 196 spin units) and 26-city (625 spin units). We compared our results with existing heuristic (Lin-Kernighan) algorithms^12^ used in the field of computer science and achieved 100% accuracy for 15-city.

## Ising formulation of Traveling Salesman Problem

Ising model, although originally introduced as a model for ferromagnetic materials, is widely applied in molecular biology, chemistry and other areas due to its combinatorial interpretation. According to this model, the spin dynamics in a ferromagnetic lattice, consisting of N lattice sites, is governed by the following Hamiltonian^2^:

1 $$H=-\sum _{\langle i,j\rangle \in neighbor}\,{J}_{i,j}{x}_{i}{x}_{j}+\sum _{i=1}^{N}\,{h}_{i}{x}_{i}$$

Here *x*_*i*_ is the spin state of the molecule at the *i*-th lattice site, which can assume either ‘up’ or ‘down’ state. *J*_*i*,*j*_ and *h*_*i*_ correspond to the energies due to the interactions with the nearest neighbors and external fields, respectively. The spins interact in such a way that they tend to eventually line up in the configuration producing the lowest value of *H*, thus transitioning from a high energy random state to a low energy ordered state at or below a critical temperature. Finding the spin configuration which minimizes H is itself an NP-hard problem. Hence, the elements of a combinatorial optimization problem can be thought of as a collection of spins, *x*_*i*_, where the Ising energy function, *H* represents the parameter to be optimized.

For example, the traveling salesman problem, which asks for the ordering of cities to visit so that the total distance travelled is the minimum, can be formulated as an Ising energy function in the following way^13^:

2 $$\begin{array}{rcl}H & = & \mathop{\underbrace{A\,\sum _{v=1}^{n}\,{(1-\sum _{j\mathrm{=1}}^{N}{x}_{v,j})}^{2}+A\,\sum _{j=1}^{N}\,{(1-\sum _{v\mathrm{=1}}^{n}{x}_{v,j})}^{2}+\sum _{(uv)\notin E}\,\sum _{j=1}^{N}\,{x}_{u,j}{x}_{v,j+1}}}\limits_{{H}_{A}}\\  &  & +\mathop{\underbrace{B\,\sum _{(uv)\in E}\,{W}_{uv}\,\sum _{j=1}^{N}\,{x}_{u,j}{x}_{v,j+1}}}\limits_{{H}_{B}}\end{array}$$

**Figure 1:**
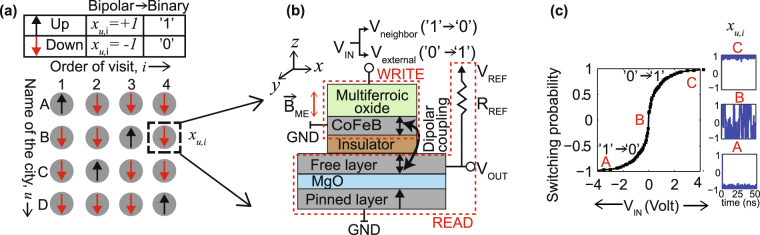
(**a**) An *N* by *N* network of spins to represent an *N* city traveling salesman problem (here *N* = 4). Each spin, *x*_*u*,*i*_ denotes whether city *u* should be visited at order *i*, where the rows represent the cities and the columns stand for the order of visit. In this work, we have assigned the binary values ‘1’ and ‘0’ to ‘up’ (magnetization moment, *x*_*u*,*i*_ = +1) and ‘down’ (*x*_*u*,*i*_ = −1) spin states, respectively. This conversion from bipolar to binary variables is shown in the table. (**b**) The geometry of the device representing each spin unit. Input voltage, *V*_*IN*_ is applied across a multiferroic oxide/CoFeB heterostructure, labeled as the ‘Write’ unit. The magnetization moment of this CoFeB layer depicts *x*_*u*,*i*_ which can point along +z (‘up’) or −z (‘down’) direction (easy axes) under the magnetoelectric effect. A voltage divider circuit, which is the ‘Read’ unit, consists of a resistance, *R*_*REF*_ and an MTJ. This ‘Read’ unit is electrically separated from the oxide/nanomagnet stack, but are magnetically coupled by dipolar interaction. The voltage across the MTJ, *V*_*OUT*_ changes depending on the state of *x*_*u*,*i*_. (**c**) The switching probability curve of the CoFeB layer vs *V*_*IN*_. The magnetization moment vs time plots are shown in the insets at positive (*C*), negative (*A*) and zero (*B*) input voltages.

For an *N* city TSP, the system consists of *N* ^2^ bits/spins *x*_*u*,*i*_ (Fig. 1(a)), which can take either ‘0’ (down) or ‘1’ (up) depending on the fact whether city *u* will be travelled at order *i* or not. *W*_*uv*_ is the distance between cities *u* and *v*. The first three terms in equation 2 consist the Hamiltonian cycle *H*_*A*_ and the last term *H*_*B*_ contains the weight/distance matrix. At the ground state of the system, *H*_*A*_ equals 0 and *H*_*B*_ denotes the minimum distance travelled. Hence, at this state, the collection of *x*_*u*,*i*_ gives us the order of cities to visit for achieving minimized H.

## Nanomagnet-based implementation

The fundamental building blocks of the Ising model, also known as artificial spin units, act as a random number generator (RNG) (randomly switches between ‘up’ and ‘down’ states) unless acted upon by external force or neighboring interactions. The stochastic switching characteristic of nanomagnets, arising from the inherent thermal noise, enables its use as RNGs. Nanomagnet-based RNGs have been demonstrated previously, like spin transfer torque based spin-dice^14^, spin-orbit torque based spin-dice^15^, voltage controlled spin-dice^16^ etc. However, high current requirement in spin-torque-based devices and unipolar switching^17^ of VCMA-based devices have stirred the quest for new mechanisms, like the magnetoelectric (ME) effect.

### Device description

Among different ferromagnet/ME oxide heterostructures^18^, *BiFeO*_3_(BFO)/CoFeB is found to demonstrate exchange bias coupling, which is suitable to accomplish 180° switching^19, 20^. Exchange bias interaction occurs at the interface between the ME oxide and the ferromagnet, while the spin and charge polarization of the multiferroic material BFO are coupled to each other. Hence, by switching the electric polarization of BFO, the magnetization of CoFeB can be switched. In our model, we have used an ultrathin CoFeB film (thickness of 0.9 nm) with perpendicular magnetic anisotropy (PMA) as the ferromagnet in contact with a multiferroic layer *BiFeO*_3_ (BFO) which consists the write unit (Fig. 1(b)). Each spin *x*_*u*,*i*_ is represented by the magnetization moment of CoFeB. The application of an electric field, *V*_*IN*_ across the ME oxide creates an effective magnetic field, ${\overrightarrow{B}}_{ME}$ experienced by *x*_*u*,*i*_. Magnetization reversal of *x*_*u*,*i*_ takes place depending on the strength and direction of ${\overrightarrow{B}}_{ME}$. Electrical switching of this type of PMA CoFeB/BFO system has been experimentally demonstrated in ref. 20. However, since the crucial aspects of these fields, like coupling mechanisms, switching dynamics etc. are still under intense research, instead of following any particular experiment set, we have used a generic parameter called magnetoelectric coefficient^21^ (*α*_*ME*_) to model the ME effect in our device. (The details of the simulation methodology is explained in the next section.) The thickness of the BFO layer is 5 nm to inhibit tunneling current in the write unit. The area of the CoFeB layer used here is small enough (16 nm × 8 nm) to make it work as an RNG when unbiased. Figure 1(c) demonstrates the switching probability of *x*_*u*,*i*_ vs input voltage, *V*_*IN*_, generated by stochastic LLG simulation. Positive values of *V*_*IN*_ creates effective ${\overrightarrow{B}}_{ME}$ in the +*z*-direction, favoring switching of *x*_*u*,*i*_ from down (‘0’) to up direction (‘1’). ${\overrightarrow{B}}_{ME}$ reverses for negative *V*_*IN*_. When *V*_*IN*_ = 0, *x*_*u*,*i*_ randomly switches between ‘up’ and ‘down’ with a lifetime of couple of nano seconds.

**Figure 2:**
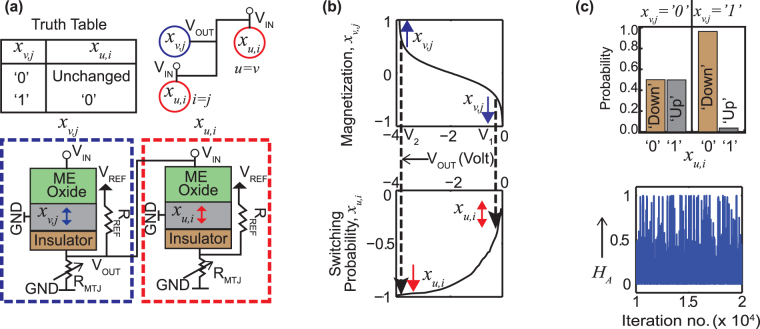
(**a**) The truth table and the physical connection governing the interactions between spin units. Here *x*_*v*,*j*_ acts as the input to *x*_*u*,*i*_ and they lie either on the same row or same column. The MTJ in each device has been replaced by a variable resistance *R*_*MTJ*_. (**b**) Top panel: the variation of *V*_*OUT*_ with *x*_*v*,*j*_, obtained from a behavioral model (*V*_*OUT*_ = *A* tanh (*Bx*_*u*,*i*_)). Bottom panel: the switching probability of *x*_*u*,*i*_ vs applied voltage, *V*_*IN*_ (stochastic LLG simulation). The polarity of this voltage tends to switch *x*_*u*,*i*_ from ‘1’ to ‘0’. (**c**) Top panel: a sample simulation of *x*_*u*,*i*_ for two cases: *x*_*v*,*j*_ = ‘0’ and ‘1’. Bottom panel: A plot of the Hamiltonian cycle, *H*_*A*_ with time. *H*_*A*_ is concentrated around 0, since the interactions are designed to make each product term *x*_*v*,*j*_ *x*_*u*,*i*_ = 0.

The read unit (Fig. 1(b)) contains a voltage divider circuit consisting of an MTJ and a reference resistance, *R*_*REF*_. This read circuit is electrically isolated from the CoFeB/BFO stack by an insulating layer. However, dipolar interaction couples the free layer of the MTJ to the magnetization *x*_*u*,*i*_ of the CoFeB layer in the write unit. The resistance of the MTJ, *R*_*MTJ*_ varies between parallel (*R*_*P*_) and antiparallel (*R*_*AP*_) configuration depending on the value of *x*_*u*,*i*_ and the output voltage, *V*_*OUT*_ changes accordingly. ${V}_{OUT}={V}_{REF}\frac{{R}_{MTJ}}{{R}_{MTJ}+{R}_{REF}}$. Here, *V*_*REF*_ denotes the supply voltage. The variation of *V*_*OUT*_ with the magnetization moment is demonstrated in Fig. 2(b), generated by a behavioral model (explained in the next section). It is to be noted that *V*_*REF*_ should not exceed the switching threshold of the MTJ free layer to keep its state unperturbed. At the same time, it should be sufficient to let *V*_*OUT*_ reach the desired values to drive subsequent units. Hence, optimization of the MTJ dimensions (i,e, *R*_*MTJ*_), *R*_*REF*_ and *V*_*REF*_ is necessary. It is worth mentioning here that the electrical isolation of the READ and WRITE unit demands two free layers for each section. It helps prevent any undesired perturbation of the current magnetization state of the WRITE free layer by the READ current.

### Neighboring and external interactions

The input voltage *V*_*IN*_ consists of two components, *V*_*neighbor*_ and *V*_*external*_ which model the interaction with neighboring units and external sources, respectively. In other words, *V*_*neighbor*_ and *V*_*external*_ are associated with the two parts of the Ising Hamiltonian (Equation 2): *H*_*A*_ and *H*_*B*_, respectively.

*H*_*A*_ imposes the restriction that each city should be visited only once. It can be reformulated into the following form:

3 $${H}_{A}\propto -\sum \,{x}_{v,j}{x}_{u,i},\quad where\,u=v\,or\,i=j$$

Each term in *H*_*A*_ contains pairs of spins lying on the same row or same column of the *N* by *N* system. Note that, the rows indicate individual cities and the columns represent their order of travel. If a spin at row *v* and column *j* is selected (*x*_*v*,*j*_ = ‘1’), all other spins in the same row (*x*_*v*,*i*_) and column (*x*_*u*,*j*_) must be ‘0’, which implies that city *v* can appear only once in the cycle and it is impossible to visit two cities *v* and *u* simultaneously. On the other hand, when *x*_*v*,*j*_ = ‘0’, *x*_*u*,*i*_ remains unchanged and no restriction is violated. In that case, *x*_*u*,*i*_ is decided by the other part of the Hamiltonian associated with the weight matrix. The truth table in Fig. 2(a) is constructed based on this principle. *V*_*OUT*_ from *x*_*v*,*j*_ is employed to the input of *x*_*u*,*i*_ in order to implement the truth table shown, with the target of making *H*_*A*_ = 0 for each term (i.e., *x*_*u*,*i*_ *x*_*v*,*j*_ = 0). The top panel in Fig. 2(b) represents how *V*_*OUT*_ changes as the magnetization moment *x*_*v*,*j*_ goes from down (*x*_*v*,*j*_ = −1 or ‘0’) to up (*x*_*v*,*j*_ = +1 or ‘1’). The corresponding switching probability is demonstrated in the bottom panel of this figure. When *x*_*v*,*j*_ points down (‘0’), the dipolar coupling causes *R*_*MTJ*_ = *R*_*P*_. Hence, the voltage divider circuit outputs a lower voltage *V*_1_ corresponding to a switching probability much less than 50% which tends to keep *x*_*u*,*i*_ unchanged (Row 1 of the truth table). On the other hand, *x*_*v*,*j*_ = ‘1’ (up) makes *R*_*MTJ*_ = *R*_*AP*_, and therefore, *V*_*OUT*_, being the voltage across *R*_*MTJ*_, equals a higher value *V*_2_ giving rise to a higher switching probability for *x*_*u*,*i*_ from ‘1’ to ‘0’ (Row 2 of the truth table). We have performed a sample simulation of two bits to validate this model. Keeping *x*_*v*,*j*_ predetermined to ‘0’ or ‘1’, we have solved stochastic LLG equation for *x*_*u*,*i*_ with *V*_*IN*_ originating from *x*_*v*,*j*_. Each simulation is performed for 100 ns, while the state of *x*_*u*,*i*_ is read after each 0.02 ns. The result is shown in the top panel of Fig. 2(c). Equal percentage in the occurrence of *x*_*u*,*i*_ = ‘0’ and *x*_*u*,*i*_ = ‘1’ is observed when *x*_*v*,*j*_ is kept fixed at ‘0’. On the other hand, *x*_*u*,*i*_ = ‘0’ dominates for input state of *x*_*v*,*j*_ = ‘1’. As expected, *H*_*A*_ is concentrated at 0 during the course of time (Fig. 2(c): bottom panel).

The other component of the input voltage *V*_*external*_ depends on *H*_*B*_ which can be re-written in the following form:

4 $${H}_{B}\propto \sum \,{W}_{u,v}{x}_{v,j}{x}_{u,i},\quad where\,j=i\pm 1$$

**Figure 3:**
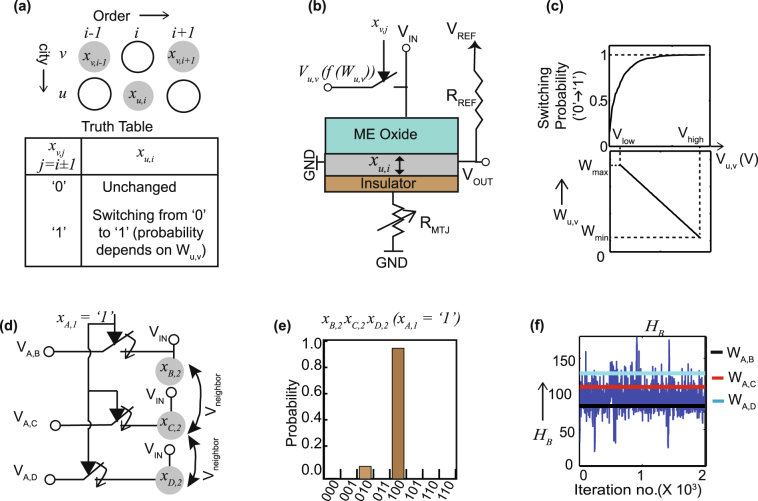
(**a**) The truth table and the position of the spin units involved in each term of *H*_*B*_, which governs the minimization of the traveling distance. (**b**) The physical realization of each term in *H*_*B*_ to mimic the truth table. (**c**) Bottom panel: linear dependence of the values of the voltage sources, *V*_*u*,*v*_ with the distance matrix, *W*_*u*,*v*_ $({V}_{u,v}={V}_{high}-\frac{{V}_{high}-{V}_{low}}{{W}_{max}-{W}_{min}}({W}_{u,v}-{W}_{min}))$. Top panel: The switching probability curve with applied voltage. The polarity of this external voltage, *V*_*u*,*v*_ tends to switch *x*_*u*,*i*_ from ‘0’ to ‘1’ (opposite of *V*_*neighbor*_). (**d**) The physical connection for a 4-city (*A*,*B*,*C*,*D*) problem with sample Hamiltonian, *H*_*B*_ = *W*_*A*,*B*_ *x*_*A*,1_ *x*_*B*,2_ + *W*_*A*,*C*_ *x*_*A*,1_ *x*_*C*,2_ + *W*_*A*,*D*_ *x*_*A*,1_ *x*_*D*,2_. City *A* is predetermined to be visited first. (**e**) The results from a coupled LLG simulation of *x*_*B*,2_, *x*_*C*,2_ and *x*_*D*,2_, keeping *x*_*A*,1_ fixed to ‘1’. ‘100’ is observed to have the highest probability of occurrence, implying that AB is the favorable path. (**f**) The Hamiltonian, *H*_*B*_ vs time, with *W*_*AB*_, *W*_*AC*_ and *W*_*AD*_ plotted in the same graph. *H*_*B*_ is concentrated near the minimum distance (*W*_*AB*_), as expected.

Each term in *H*_*B*_ dictates whether city *u* and *v* should be visited consecutively depending on the distance between them, *W*_*u*,*v*_. At the ground state of the system, *H*_*B*_ refers to the minimum distance travelled. With this restriction in mind, the truth table in Fig. 3(a) works towards selecting the spins in consecutive columns based on their distances. Note that, in our model, a spin *x*_*v*,*j*_ in state ‘1’ implies that city *v* is chosen to visit at order *j*. Each spin *x*_*v*,*j*_ from Equation 4 is situated at (*i* + 1)-th or (*i* − 1)-th columns, whereas *x*_*u*,*i*_ lies at the *i*-th column (Fig. 3(a)), given the columns dictate the order of travel. Each term in Equation 4 is modeled as an external input voltage *V*_*u*,*v*_ being applied to the spin unit *x*_*u*,*i*_, while this voltage source is switched ‘ON’/‘OFF’ by the states of the spin units *x*_*v*,*j*_ in the adjacent columns (Fig. 3(b)). In case of the last column (column *N*), the adjacent columns are (*N* − 1)*th* and 1*st* columns, respectively considering a closed loop. The value of *V*_*u*,*v*_ is set by the *W*_*u*,*v*_ vs *V*_*u*,*v*_ curve shown in the bottom panel of Fig. 3(c). At smaller *W*_*u*,*v*_, *V*_*u*,*v*_ is set to a large value, *V*_*high*_ which favors the switching from ‘0’ to ‘1’ and vice versa. Figure 3(d–f) show the results from the simulation of a sample Hamiltonian, *H*_*B*_ = *W*_*A*,*B*_ *x*_*A*,1_ *x*_*B*,2_ + *W*_*A*,*C*_ *x*_*A*,1_ *x*_*C*,2_ + *W*_*A*,*D*_ *x*_*A*,1_ *x*_*D*,2_ for a 4-city (*A*,*B*,*C*,*D*) TSP. Note that the first letters in the subscript of the variable names (*x*_*A*,1_ etc.) refer to the city names, whereas the second letters denote the order of visit for that city. Three coupled stochastic LLG equations are solved for *x*_*B*,2_, *x*_*C*,2_ and *x*_*D*,2_ with external inputs *V*_*A*,*B*_, *V*_*A*,*C*_ and *V*_*A*,*D*_, respectively (Fig. 3(d)). Hence, we are trying to determine which city among *B*,*C* and *D* should be visited at order 2, given city *A* is visited first (*x*_*A*,1_ = 1). The switches are always ‘ON’ (because *x*_*A*,1_ = ‘1’). In addition, all units are being injected with *V*_*neighbor*_, as described in the previous paragraph, to prevent simultaneous selection of spins in the same column. In this simulation, we have chosen *W*_*A*,*B*_ < *W*_*A*,*C*_ < *W*_*A*,*D*_. Therefore, Fig. 3(e) demonstrates that ‘100’ is the favorable state for *x*_*B*,2_ *x*_*C*,2_ *x*_*D*,2_, implying that city *A* to *B* is the favorable path, not *A* to *C* or *A* to *D*. Also, *H*_*B*_ is centered at *W*_*AB*_ (Fig. 3(e)), which is the shortest route.

## Simulation Methodology

**Figure 4:**
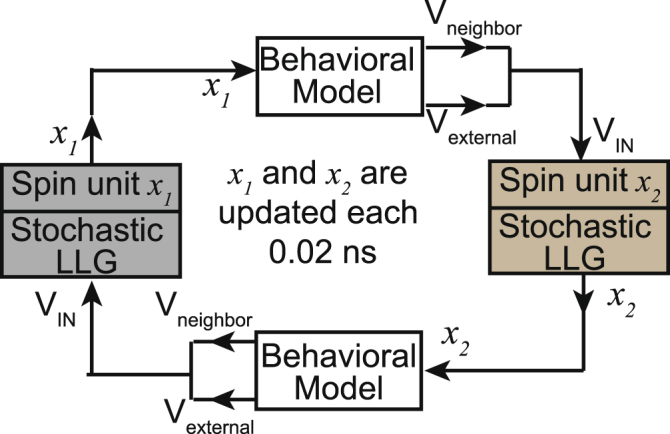
A schematic showing the flow-diagram of our coupled simulation framework for two sample spin units *x*_1_ and *x*_2_. This cycle is performed for all of the (*N* − 1)^2^ spin units.

In order to solve an *N* by *N* problem, we have used an *N* − 1 by *N* − 1 network of spins keeping node 1 fixed to appear first in the cycle. To calculate the magnetization dynamics of these (*N* − 1)^2^ nanomagnets, we have developed a simulation framework consisting of a set of (*N* − 1)^2^ stochastic LLG equations coupled with each other through their input voltages, *V*_*IN*_. In our numerical simulation, we have used a time step of 0.02 ns. After each time step, the magnetization moment of each bit is updated, and these updated values are used to generate the input voltages for the next time cycle (Fig. 4). A behavioral model is used to generate the input voltages, taking corresponding spin values as inputs. The details of the micromagnetic simulation and the behavioral model are described in this section.

### Micromagnetic simulation

The LLG equation with monodomain approximations is described as follows^22^,

5 $$\frac{d\hat{m}}{dt}=-\gamma (\hat{m}\times {\overrightarrow{H}}_{eff})+\alpha (\hat{m}\times \hat{m}\times {\overrightarrow{H}}_{eff})$$

Here $\hat{m}$ is the normalized magnetization moment of the ferromagnet layer, *α* is the Gilbert damping constant, *q* is the charge of an electron and *γ* denotes the gyromagnetic ratio. ${\overrightarrow{H}}_{eff}$ is the effective magnetic field acting on the magnetization, which consists of the anisotropic field, ${\overrightarrow{H}}_{anisotropy}$, demagnetization field, ${\overrightarrow{H}}_{demag}$, thermal field, ${\overrightarrow{H}}_{thermal}$ and effective magnetoelectric field, ${\overrightarrow{H}}_{ME}$. Note that there is no spin transfer torque due to negligible current flow through the oxide.

${\overrightarrow{H}}_{anisotropy}$ and ${\overrightarrow{H}}_{demag}$ are calculated according to refs 23 and 24. Thermal field, ${\overrightarrow{H}}_{thermal}$ is given by ref. 25

6 $${\overrightarrow{H}}_{thermal}=\overrightarrow{\xi }\sqrt{\frac{2\alpha {k}_{B}T}{|\gamma |{M}_{s}Voldt}}$$

*k*_*B*_ is the Boltzmann constant, *T* is the temperature of the system, *M*_*s*_ is the saturation magnetization, *Vol* is the volume of the free layer and *dt* is the discrete time step used in the numerical simulation. $\overrightarrow{\xi }$ is a 3-component vector whose components are zero mean Gaussian random variables with standard deviation of 1.

We have modeled the ME field through the magneto-electric coefficient *α*_*ME*_^26^,

7 $${\overrightarrow{H}}_{ME}=(0\widehat{x},\,0\widehat{y},\,{\alpha }_{ME}(\frac{{V}_{IN}}{{t}_{ME}}){m}_{z}\widehat{z})$$

Note that the easy axis of the PMA magnet is along z-direction. Here *V*_*IN*_ is the voltage across the ME oxide, *t*_*ME*_ is the thickness of the oxide layer and ${\alpha }_{ME}={\mu }_{o}\frac{dB}{dE}=\frac{magnetic\,field}{electric\,field}$. The experiment in ref. 27 shows a nonlinear variation in *α*_*ME*_ versus the applied voltage, with the values ranging from ~0.001×10^−7^ *sm* ^−1^ to 1 × 10^−7^ *sm* ^−1^. However, for the voltage range in our model, we have used an average value of 0.03 × 10^−7^ *sm* ^−1^ (1/*c*, c = speed of light). The parameters used in the LLG simulations are listed in Table 1.

**Table 1:** Summary of the parameters used in the simulation.

Parameters	Values
CoFeB layer dimension	16 nm × 8 nm × 0.9 nm
Damping constant, *α*	0.15
Saturation magnetization, *M*_*s*_	250 emu
Interface anisotropy, *K*_*i*_	0.068 *mJ*/*m* ^2^
Magneto-electric coefficient, *α*_*ME*_	0.03 × 10^−7^ *s*/*m*
ME oxide thickness	5 nm

### Behavioral model

In order to calculate *V*_*IN*_ (in Equation 7) applied to each ME oxide/nanomagnet unit, we have used behavioral models to calculate the constituents *V*_*neighbor*_ (Fig. 2(b)) and *V*_*external*_ (Fig. 3(b)) which require relevant spins, *x*_*u*,*i*_/*x*_*v*,*j*_ (generated by LLG equations) as inputs. Since *R*_*MTJ*_ follows a sigmoid function with its magnetization moment (SPICE model of the MTJ: ref. 29), *V*_*neighbor*_ (i.e., *V*_*OUT*_, which is linearly dependent on *R*_*MTJ*_) is modeled as *A* tanh (*Bx*_*u*,*i*_), where the parameters *A* and *B* are adjusted to fit the desired values of *V*_1_ and *V*_2_ (Fig. 2(b)). On the other hand, *V*_*external*_ is linearly related to the distance matrix by the equation: ${V}_{external}={V}_{high}-\frac{\delta V}{\delta W}(W-{W}_{min})$, when corresponding control spins *x*_*v*,*j*_ = ‘1’. Slope $\frac{\delta V}{\delta W}=\frac{{V}_{high}-{V}_{low}}{{W}_{max}-{W}_{min}}$. Here *W* is the value of the corresponding weight/distance, *W*_*min*_ and *W*_*max*_ are the minimum and the maximum value in the distance matrix, respectively. This behavioral model and the set of coupled LLG equations are solved self-consistently with a time step of 0.02 ns.

In addition, we have used simulated annealing in order to gradually move towards the global minima. The total time required for the nanomagnet array to reach a steady state starting from a random distribution is around 10 *μ*s with an annealing period of 50 ns. At *i*-th period, |*V*_2,*i*_| = |*V*_2,*initial*_| + *i* × *δV*_*neighbor*_, |*V*_*low*,*i*_| = |*V*_*low*,*initial*_| + *i* × *δV*_*external*_ and |*V*_*high*,*i*_| = |*V*_*high*,*initial*_| + *i* × *δV*_*external*_. (*V*_2_ is shown in Fig. 2(b), *V*_*low*_ and *V*_*high*_ are shown in Fig. 3(c)). In this way, we move towards a steady state as time goes by. The efficiency of the annealing schedule largely depends on the initial points and the cooling rate. In our simulation, we have used |*δV*_*neighbor*_| = 50 *mV* and |*δV*_*external*_| = 100 *mV*.

## Results

**Figure 5:**
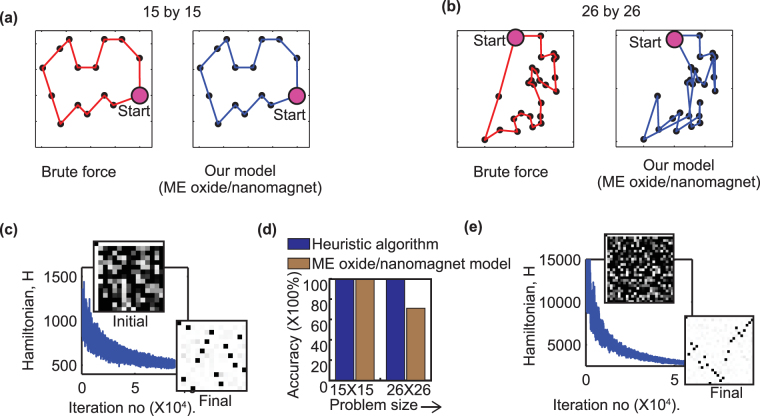
The travel route obtained from brute force search^28^ (Red) and our simulation (Blue) for (**a**) 15-by-15 and (**b**) 26-by-26 traveling salesman problem. The start city is identified with a bigger pink circle in each route. The plot of the Ising Hamiltonian with time for (**c**) 15-by-15 and (**e**) 26-by-26 problems. The initial and final magnetization states of the *N* by *N* networks are shown in the insets. (**e**) A bar chart comparing our simulation result with LK heuristic algorithm^12^.

We developed a coupled LLG equation-behavioral model simulation framework to solve TSPs of two sizes: 15 by 15 ((*N* − 1)^2^ = 196 nodes) and 26 by 26 (625 nodes). Figure 5(a,b) show a side-by-side comparison of the routes obtained by brute force search (red) and our approach based on ME oxide/nanomagnet stochastic device (blue), where the case for 15-by-15 matches 100%. However, as the problem size increases, the solution becomes more susceptible to the efficiency of the annealing schedule. Hence, the accuracy goes down for the 26 by 26 problem. However, the aim of our work is not to analyze optimized annealing schedule, rather demonstrate that near-optimal solution is achievable with this ME oxide/nanomagnet based stochastic device. The problem dataset and brute-force solutions are taken from refs 28 and 30. The plots of the Hamiltonian function in Fig. 5(c,e) indicate that the system moves towards low energy ordered state over time as we approach solution. The initial and final magnetization states are shown in the insets. Figure 5(d) shows a comparison of our results with LK^12^ heuristic algorithm. It is possible to boost up the accuracy of the 26-city problem by adjusting the annealing schedule. The code for solving the LK algorithm with our test data has been taken from ref. 31.

We have also calculated the amount of energy dissipation in the spin units. As stated earlier, current flow in the write unit and the interconnect is negligible. Hence, power dissipation takes place only in the read unit. With the aim of making *V*_*OUT*_ sufficient (~2 V) to provide *V*_*IN*_ to coupled spin units without any intermediate amplification, we have used *V*_*REF*_ = 2.5 *V*, *R*_*REF*_ = 25 *k*Ω. *R*_*MTJ*_ varies between 15 kΩ (*R*_*p*_) and 40 kΩ (*R*_*ap*_) (SPICE simulation). Note that the area of the MTJ is large (96 nm × 72 nm) enough to make the critical switching current much larger (high energy barrier) than the current flow in the voltage divider circuit. With these parameters, the maximum power dissipated in the individual spin units amount to 0.15 mW. It can be reduced by using better multiferroic oxides with strong magneto-electric effect, i.e. large magnetoelectric coefficient *α*. To this date, the value of *α* (at low voltages) obtained experimentally for exchange bias effect is limited to 1/c to 0.01/c, c is the speed of light^19^. However, if ME oxides with *α* ≥ 5/*c* is found, *V*_*OUT*_ can be reduced to 0.5 V (obtained from stochastic LLG simulation). Hence, *V*_*REF*_ ≤ 1 *V* and power dissipation in individual units ≤0.025 *mW* can be achieved.

## Discussion

In conclusion, we have presented a theoretical demonstration of a magnetoelectric oxide based stochastic spin unit to model the Ising Hamiltonian of traveling salesman problem. It operates based on voltage-controlled switching of ferromagnets instead of current. The core of the unit is a thick multiferroic oxide/nanomagnet stack which permits negligible current flow, making the scheme energy-efficient and easy to scale, unlike other current-based units. Aside from reduced current flow, the voltage based coupling mechanisms make the routing network less prone to leakage. Using our coupled micromagnetic simulation framework, we have demonstrated simulation results for problems as large as 625 nodes, with 100% accuracy for 15-city (196 nodes) problems. Moreover, we have provided guidelines towards reducing power dissipation without the need of any intermediate amplification between cascaded spin units. However, some challenges, like dielectric breakdown of the ME oxide along with ways to overcome them are left for future investigation.
